# Supplementary material for: Practical guidance for firefighter applicants preparing for cardiorespiratory fitness testing: a secondary analysis of self-reported physical activity levels
Source: PeerJ. 2022 Sep 5;10:e13832. doi: 10.7717/peerj.13832 (PMC9455017; doi:10.7717/peerj.13832)
Supplement: Supplemental Information 1 — Physical activity readiness questionnaire (PAR-Q) [file peerj-10-13832-s001.pdf]

MEMORIAL UNIVERSITY

ALLIED HEALTH SERVICES
SCHOOL OF HUMAN KINETICS AND RECREATION

PHYSICAL ACTIVITY READINESS QUESTIONNAIRE (PAR-Q)

Your comments and responses on the questionnaire are confidential and will be handled according to the ethical guidelines of the Canadian Society of Exercise Physiology (CSEP)

Name: ____

Address:

Street:____

City/Province:____

Postal Code:____

Phone: ____

Email: ____

Date of Birth: ____

Age: ______________________________ Sex: _______________________________

Height: ____________________________ Weight: ____________________________

Occupation: ____

Emergency Contact Information:

Name:____

Phone:__________________________ Relationship:_____________________________

Signature: __________________________ Date:________________________________

1. RISKS ASSOCIATED WITH CARDIOVASCULAR PROBLEMS

	YES	NO
1.1 How long ago since you have seen a doctor?		
- less than 6 months	☐	☐
- 6 to 18 months (half a year to a year)	☐	☐
- more than 18 months (year and a half)	☐	☐
1.2 Has your doctor ever told you that you have a heart problem?	☐	☐
1.3 Do you experience chest or heart pains frequently?	☐	☐
1.4 Do you experience dizziness or weakness frequently?	☐	☐
1.5 Do you ever have palpitations, that is, your heart beating more rapidly for no reason?	☐	☐
1.6 Do you have bad headaches?	☐	☐
1.7 Are your ankles and/or knees often swollen (without being a result of an injury)?	☐	☐

Comments:

____

____

____

____

____

2. RESPIRATORY SYSTEM

	YES	NO
2.1 Do you suffer from:		
- asthma	☐	☐
- bronchitis	☐	☐
- emphysema	☐	☐
- other respiratory sickness	☐	☐
- sleep apnea	☐	☐

*Specify*__________________________________

3. METABOLIC PROBLEMS

	YES	NO
3.1 Within the past six months has your doctor said to you that:		
-you have hypertension	☐	☐
-you have type II diabetes	☐	☐
-you have impaired glucose tolerance	☐	☐
-you have hypercholesterolemia	☐	☐
-you have high triglycerides	☐	☐
-you have low HDL	☐	☐
-you are obese	☐	☐

*Others:*____

4. LOCOMOTOR SYSTEM

	YES	NO
4.1 Has your doctor ever told you that you have bone or joint problems (such as arthritis for example) which might be worsened through exercise?	☐	☐
4.2 Do you suffer from:		
-frequent back pain	☐	☐
-hernia	☐	☐
-spinal cord problems/vertebral column problems	☐	☐
-problems with your meniscus (joints)	☐	☐
-ligament problems	☐	☐
-tendon problems	☐	☐

*Comments:*____
____
____

5. RISKS ASSOCIATED WITH FAMILY PREDISPOSITION

	YES	NO
5.1 Are there any members of your family (parents, grandparents, brothers, sisters, aunts, uncles) who have:		
- suffered from chronic high blood pressure	☐	☐
*if yes, how many member(s)*_____________		
- suffered from adult-onset diabetes	☐	☐
*if yes, how many member(s)*_____________		
- suffered from juvenile diabetes	☐	☐
*if yes, how many member(s)*_____________		
- died from a heart attack before the age of 60	☐	☐
*if yes, how many member(s)*_____________		

*Comments:*____
____
____

6. NEUROLOGICAL PROBLEMS

	YES	NO
6.1 Have you ever had a head injury or concussion?	☐	☐
- were you treated by a physician?	☐	☐
- were you hospitalized?	☐	☐

if yes, when ______________________

- what were your symptoms? ____
- how long did they last? ____

	YES	NO
6.2 Have you ever become unconscious or lost your memory?	☐	☐
6.3 Have you ever had a seizure?	☐	☐
6.4 Do you have frequent or severe headaches?	☐	☐

	YES	NO
6.5 Have you ever had tingling into your arms, hands, legs, or feet?	☐	☐
6.6 Have you ever had a nerve injury?	☐	☐

7. <u>OTHERS/MISCELLANEOUS</u>

	YES	NO
7.1 Have you had a major surgical operation within the past two years, or an illness requiring a stay in the hospital?<br>*if yes, specify:* ______________________________	☐	☐
7.2 Are you still suffering the operation or illness?	☐	☐
7.3 Do you feel ill?	☐	☐
7.4 Are there other physical reasons which would prevent you from following a physical exercise program even if you wanted to do so?<br>*if yes, specify:* ______________________________	☐	☐

8. <u>PHYSICAL ACTIVITY AND EXERCISE HABITS</u>

	YES	NO
Have you been physically active for one year or more?	☐	☐

If yes, indicate the number of <u>minutes per week</u> currently performing for each activity on the next page:

ACTIVITIES	MINUTES/WEEK	ACTIVITIES	MINUTES/WEEK
Dance	_______	Racquetball	______
Folk Dance	_______	Tennis	______
Canoeing	_______	Kayaking	______
Climbing	_______	Martial Arts/Combative	______
Squash	_______	Basketball	______
Baseball/Softball	_______	Volleyball	______
Soccer	_______	Running	______
Hockey	_______	Ice Skating	______
Badminton	_______	Bicycling	______
Volleyball	_______	Swimming	______
Curling	_______	Cross-Country Skiing	______
Stationary Bicycle	_______	Downhill Skiing	______
Jump Rope	_______	Diving	______
Brisk Walking	_______	Rollerblading	______
Rowing	_______	Sailing	______
Wrestling	_______	Handball	______
Snowshoeing	_______	Weight Training	______

If the activities that you practice are not indicated in the list, please list them below and indicate the number of minutes a week.

ACTIVITIES	MINUTES/WEEK

Athletes:

Are you currently:

- -resting Number of week(s): _________
- -beginning training Number of week(s): _________
- -competing Number of week(s): _________
- -ill Number of week(s): _________

*Comments:*____

____

____

9. TOBACCO/TOXINS

	YES	NO
9.1 Do you smoke?	☐	☐
9.2 Have you ever smoked?	☐	☐

If yes for question 9.1 or 9.2:

A – Cigarettes
How many do/did you consume in a day?

10 or less ☐ 20-30 ☐
20 or less ☐ 30 or more ☐

B – Pipe/Cigars
How often do/did you smoke pipes/cigars?

Occasionally ☐ Regularly ☐

C – For how many year(s) have you been smoking (or for how many years did you smoke)?

Less than a year ☐ 2-5 years ☐ More than 5 years ☐

D – If you used to smoke but don't smoke any longer, how many year(s) since you stopped?
__________ year(s)

10. DIETARY HABITS

	YES	NO
10.1 Do you make time to eat breakfast each morning?	☐	☐
10.2 Is it important for you to consume three meals each day?	☐	☐
10.3 Have you ever been instructed by a dietitian or receive nutritional counseling from another health care providers?	☐	☐

if *yes, when*? ________________________________

	YES	NO
10.4 Have you ever followed a diet program in the past?	☐	☐

if yes, when and what program? ____

____

____

10.5 How many cup(s) of caffeine beverages (coffee and/or tea) do you consume each day?

2 or less ☐ 3-5 ☐ 6 or more ☐

10.6 Indicate the number of alcoholic beverage(s) you consume each week (beer, wine, spirits)?

Never Have ☐ Quit ☐ 1-2 ☐
3-6 ☐ 7-24 ☐ 25+ ☐

11. **STRESSORS**

	YES	NO
11.1 Is your profession or work liable to increase, in a potentially harmful way, your stress levels and make you irritable?	☐	☐
what is your profession/work? __________________________		
11.2 Do you have difficulty falling asleep or sleeping uninterrupted for at least 6 hours?	☐	☐
11.3 What is your average daily number of hours of sleep? _________		

12. **WEIGHT**

12.1 In your opinion, is your weight 10 kg (25 lbs) or more above normal weight?	☐	☐
12.2 Do you have trouble gaining or losing weight?	☐	☐
12.3 Have you experienced any recent weight change?	☐	☐

13. **MEDICATIONS**

13.1 Do you take any medications regularly? (example: for high blood pressure)	☐	☐

if yes, specify: ____

____

____

14. **FOR WOMEN ONLY**

	YES	NO
14.1 Do you have gynecological problems which are heightened by physical activity?	☐	☐

if yes, specify: ____

____

____

14.2 Are you pregnant?	☐	☐
14.3 How many times have you been pregnant? __________		
14.4 How many full-term pregnancies have you had? ___________		
14.5 Are you presently taking oral contraceptives?	☐	☐
14.6 Have you ever taken oral contraceptives?	☐	☐

14.7 If you answered yes to questions 14.5 and 14.6, for how long do/did you use them on a regular basis? ______ year(s)

14.8 If you have already used them in the past and no longer use them, how long ago did you stop?
______ year(s)

	YES	NO
14.9 Are your menstrual cycles generally regular (between 25 and 35 days)?	☐	☐
if no, do they happen more frequently (less than 25 days between)?	☐	☐
or, do they happen less frequently (more than 35 days between)?	☐	☐
14.10 Are you menopausal?	☐	☐
if yes, for how long: _____ year(s)		
14.11 Have you experienced long periods without menstruating?	☐	☐

*Comments:*____
____
____
