# Supplementary material for: Practical guidance for firefighter applicants preparing for cardiorespiratory fitness testing: a secondary analysis of self-reported physical activity levels
Source: PeerJ. 2022 Sep 5;10:e13832. doi: 10.7717/peerj.13832 (PMC9455017; doi:10.7717/peerj.13832)
Supplement: Supplemental Information 2 — Supplemental data handling [file peerj-10-13832-s002.pdf]

Addendum 2 – Data Handling

Raw data from the GXT were analyzed in MS Excel to determine maximal physiological values at exhaustion. All data were smoothed over a 30-second moving average to determine applicants' maximal physiological values. All physical characteristics and physiological parameters were then plotted separately to detect possible abnormalities and body mass index (BMI) was calculated using the height and the body mass of each applicant.

The data from the section pertaining to physical activity and exercise habits was used to calculate and to plot total weekly activities performed by each applicant. Since the PAR-Q was filled out in February, all activities that could only be performed in the summer in Newfoundland and Labrador were removed on the basis that applicants did not answer the questionnaire correctly, that is, to only include activities currently performing on a regular basis. The scatter plot revealed that still five observations were showing greater than 1020 min/week which is equivalent to 17 hours of activities per week after removal of summer-only activities. Knowing that it is excessive for this population, a closer look at their occupations was performed for these five applicants. It was determined that some of them had physically active jobs but were not necessarily exercising at work. For example, it was noticed that one applicant recorded walking 32 h/week as a delivery driver. Although physical activity plays an important role in the prevention and treatment of multiple chronic diseases, we were interested in the amount of exercise applicants performed in preparation for the GXT. Physical activity and exercise are often used interchangeably, but these terms are not synonymous. Physical activity is defined as any bodily movement produced by the contraction of skeletal muscles that results in a substantial increase in caloric requirements over resting energy expenditure. Exercise is a type of physical activity consisting of planned, structured, and repetitive bodily movement done to improve and/or maintain one or more components of physical fitness (ACSM, 2021). Although ACSM recommends that adults should accumulate 30 minutes or more of moderate physical activity on most, preferably all, days of the week to maintain health-related benefits associated with being active, this recommendation is probably not suited for firefighter applicants who require to undergo at least two physically demanding tests during their recruitment process and require above average cardiorespiratory fitness level. Consequently, the parameter of interest with firefighter applicants is not physical activity but rather planned, structured exercise with the main objective to improve or maintain physical fitness. For this reason, activities considered as physical activity, such as walking and dancing, were removed. The updated scatter plot showed two observations that were still relatively high in total exercise training time, but were kept in the dataset for further analysis of outliers.

Finally, all activities were divided into two categories: endurance exercise and strength and power exercise. Also called aerobic exercise, the term endurance exercise training generally refers to training the aerobic system and includes activities that increase your breathing and HR. From the PAR-Q, 31 activities were considered endurance-based (e.g., running, biking, hockey, etc.) whereas three were strength and power activities (resistance training, downhill skiing, and martial arts). Strength and power exercise are commonly called resistance training, weight training, or power training, and improves muscular strength. For the remaining of the document, strength and power exercises will be referred to strength exercise for ease of readability. The sum of all endurance exercises and all strength exercises in min/week were calculated for each applicant. The total exercise training was the addition of self-reported endurance exercises and self-reported strength exercises which describes the total training time or weekly training volume.

Additional data inspection and cleaning were performed using Python 3 (Python Software Foundation, Delaware, USA). The data were checked for duplicates, negatives, and missing values. HR recovery,

$\dot{V}CO_2$, $\dot{V}_E$, breathing frequency, and RER were missing two values. Knowing that physiological parameters are specific to each individual, they were not replaced by another value. However, the missing values for HR recovery were replaced by the mean from their group (successful vs. unsuccessful). Mean values along with the maximal and minimal values for all parameters were then inspected for abnormalities. Outliers were investigated by graphing a boxplot for each parameter to visually identify potential outliers. According to the graphs, there were potential outliers in all parameters when it was not the case from the preliminary data exploration. Thus, it was decided to try another method to detect outliers mathematically this time using the *z*-score. The standard cutoff value of ±3 for finding outliers was used. Nine observations in six different parameters were detected as potential outliers. After looking specifically at each of them, it was decided that outliers for age, body mass, and Abs$\dot{V}O_2$ were all possible values and should remain in the dataset. On the other hand, extreme values with *z*-scores up to seven for endurance exercise, strength exercise, and total exercise were considered not realistic for this population and were consequently dropped. One possible explanation could be once again the misunderstanding of how to fill out the physical activity and exercise habits section of the questionnaire. The question was to indicate the number of minutes currently performing each activity on a weekly basis. For applicants with extreme values, it looked like all activities performed throughout the year were entered instead of only those currently performed each week. In return, it created outliers for endurance exercise training time up to 26 h/week, strength exercise training time up to 12 h/week, and total exercise training time up to 28 h/week, which were all removed from the dataset as previously mentioned.
