# Supplementary material for: Practical guidance for firefighter applicants preparing for cardiorespiratory fitness testing: a secondary analysis of self-reported physical activity levels
Source: PeerJ. 2022 Sep 5;10:e13832. doi: 10.7717/peerj.13832 (PMC9455017; doi:10.7717/peerj.13832)
Supplement: Supplemental Information 3 — Supplemental statistical analyses [file peerj-10-13832-s003.pdf]

Addendum 3 – Statistical Analysis

The complete dataset was divided into two groups, successful group (SG) and unsuccessful group (UG) to assess if there was a statistical difference between the two groups. Two-sample *t*-test was conducted using Python 3 (Python Software Foundation, Delaware, USA) to test the equality of two means from independent populations. Normality was inspected by observing the histograms and by conducting the Shapiro-Wilk test which showed that most parameters, except for age and body mass, followed a normal distribution. However, since the central limit theorem states that the distribution of sample means approximates a normal distribution as the sample size gets larger than 30, normality was assumed for all parameters used for calculating the *t* statistic. Homogeneity of variances was tested using the Levene's test and all parameters failed to prove equal variances between groups. This resulted in using Welch's *t*-test which provides a better control of Type I error rates when the assumption of homogeneity of variance is not met.

Subsequently, the original dataset was divided into three fitness zones according to maximal $\dot{V}O_2$ values. The red zone includes applicants with maximal $\dot{V}O_2$ values ≤ 40 ml·kg^{-1}·min^{-1}, the yellow zone includes applicants with $\dot{V}O_2$ values between 40–45 ml·kg^{-1}·min^{-1}, and the green zone includes applicants with $\dot{V}O_2$ values ≥ 45 ml·kg^{-1}·min^{-1}. A one-way Welch analysis of variance (ANOVA) was performed on all parameters of interest to assess the difference between the mean for each fitness zone and a pairwise Games-Howell post hoc test was used to compare all possible combinations of fitness zone differences.

In inferential statistics, null hypothesis significance testing only informs of the probability of the observed or more extreme data given that the null hypothesis is true (i.e., *p* value) upon which a dichotomous decision is made: reject or fail to reject the null hypothesis. However, null hypothesis significance testing does not provide what are probably the two most important pieces of information in statistical inference: (i) the estimates of the magnitude of the difference in outcomes between groups (effect size) and (ii) the precision of that estimate (confidence interval for the effect size). Standardized effect size can be grouped in two families: the *d* family (standardized mean differences) and the *r* family (measures of strength of association). Conceptually, the *d* family effect size is based on the difference between observations, divided by the standard deviation of these observations. They are signed effect size where the sign of the statistic indicates the direction of the corresponding contrast. The *r* family effect size describes the proportion of variance that is explained by group membership where the sign indicates the direction of the relation between two parameters.

Unstandardized (mean difference between groups) as well as standardized effect size (*d* statistic) are reported in this article along with their corresponding confidence interval (CI) when applicable. Not only does the width of the CI directly indicate the amount of sampling error associated with a particular effect size, it also estimates a range of effect size in the population that may have given rise to the observed result. In general, minimal effect size should be beyond the error of the measuring device to ensure differences are not due to measurement error. Cohen (1988) proposed conventional values as benchmarks for what are considered to be 'small', 'medium', and 'large' effects. A medium effect is one that is "visible to the naked eye of the careful observer", a small effect is one that is less than a medium effect, but greater than a trivial effect, and a large effect is one that is far above a medium effect. The location of the *d* statistic determines if the outcome is considered harmful, beneficial, or trivial. A positive effect size > .20 is considered beneficial, while a negative effect size < -.20 is considered harmful. Effect size between -.20 and .20 are trivial in size (Cohen, 1992). This means that if two groups' means don't differ by at least .20 standard deviations, the difference is trivial even if it is statistically significant. Unstandardized effect size (ES_{M1-M2}) was calculated by subtracting the mean of

UG from the mean of SG whereas the standardized *d* statistic effect size was calculated using the *t* statistic with the following formula (Kline, 2013; Grissom & Jim, 2011):

$$d = t * \sqrt{(n_1 + n_2 / n_1 n_2)}$$

where
$d = d$ statistic
$t = t$ statistic
n_1 = sample size SG
n_2 = sample size UG

The CI for the *d* statistic were constructed using the standard error, *t*-score, and the degree of freedom (*df*) from the *d* statistic for unequal variances as suggested by Kline, 2013.

$$CI = d +/- \sqrt{[d^2 / 2df + (n_1 + n_2 / n_1 n_2)]} * [t_{2\text{-tail}, \alpha} (df)]$$

It is common to use boxplot to display the distribution of data by graphically depicting groups trough their quartiles. In a notched boxplot, the notch is around the median and is used as a rough guide to significance of the difference of medians between groups. Upper and lower notches are the upper and lower bounds of 95% CI of the median and the distance between the notches shows the most likely values expected for the median. If upper and lower notches don't overlap, it indicates a distinct difference in the medians between groups. On the other hand, whiskers indicate the variability outside the upper and lower quartiles. The lower whisker displays the lowest data point excluding any outliers whereas the upper whisker represents the largest data point excluding any outliers. Boxplot outliers are computed using the interquartile range (IQR) method and are typically displayed as dots below and above whiskers. Since outliers in our analysis were identified using *z*-scores instead of IQR and true outliers were removed before analyzing our data, the dataset used to create boxplots were considered free of extreme values. As a result, Fig. 2 and 3 do not display outliers and upper whiskers shown on Fig. 3 exclude the maximal value for each box.
